# Factors Determining Unique Thermal Resistance and Surface Properties of Silicone-Containing Composites

**DOI:** 10.3390/ma17246088

**Published:** 2024-12-13

**Authors:** Maria Zielecka, Anna Rabajczyk

**Affiliations:** Scientific and Research Centre for Fire Protection—National Research Institute, Nadwiślańska 213, 05-420 Józefów, Poland; arabajczyk@cnbop.pl

**Keywords:** thermal resistance, surface properties, silicone-containing composite, self-cleaning

## Abstract

This review discusses the key factors influencing the exceptional thermal resistance and surface properties of silicone-containing composites. Silicone polymers, known for their excellent chemical and physical properties, are widely used in a number of innovative products. In order to make silicone composites suitable for innovative applications, it is essential to ensure that they have both very good thermal resistance and superhydrophobic properties. Identification of the key factors influencing these properties enables the use of these composites in coatings, electronics and photovoltaic panels. The discussion includes the role of organosilicon polymer structures and the incorporation of micro- and nanoadditives to enhance the performance of these materials. Different methods for the modification and production of silicone composites are analyzed, with an emphasis on achieving thermal stability and surface superhydrophobicity simultaneously. The review highlights the growing demand for silicone-based coatings due to technological advances and environmental concerns. Furthermore, the role of surface modification and hierarchical surface structures in achieving these unique properties is discussed, as well as the potential applications and challenges in the development of next-generation silicone-containing materials.

## 1. Introduction

Organosilicon polymers, popularly called silicones, have special chemical and physical properties, which gives them great potential for use in various modern, innovative products. Particularly important is the possibility of simultaneous use of such functional parameters of these polymers as very good thermal resistance and unique surface properties. Increased thermal resistance ensures the possibility of safe and long-term use of the products in a wide range of temperatures [1,2]. The unique surface properties are most often related to superhydrophobicity and a self-cleaning ability [3,4]. This set of properties means that the scale and scope of use of organosilicon polymers is constantly increasing. Examples include modern coating materials [5,6], electronic devices [7], coatings applied in medical implants [8] and photovoltaic panels [9,10,11].

Analysis of the silicone coatings market indicates continuous growth in this area. According to the Data Bridge Market Research report [12], prepared for the forecast period of 2022–2029 taking into account data from 2021 as the base year, the silicone coatings market was valued at USD 5.51 billion in 2021 and is expected to reach USD 8.72 billion by 2029, which means a compound annual growth rate (CAGR) of 5.90% during the forecast period of 2022–2029. The report was developed based on in-depth expert analysis, import/export analysis, price analysis, patent analysis and technological progress.

Slightly less optimistic data expressed in the CAGR, at the level of over 4%, but proving stable growth, were published in a report prepared by the Mordor Intelligence Research & Advisory [13]. The use of silicone coatings in the construction industry was identified as the main market drivers in this report. At the same time, the increase in demand for silicone coatings due to their unique application parameters in industries such as electronics, automotive and biomedical was also highlighted [14]. In these applications, the most commonly used coatings are the so-called conformal coatings [15,16,17], which are a specially developed polymer product that creates a coating protecting printed circuit boards, components and other electronic devices against harmful environmental conditions such as moisture, thermal shock, electrostatic charges, vibrations and contamination.

High costs of raw materials have been identified as a factor inhibiting the development of the market, which is most important in mass applications and less significant in applications in innovative products. At the same time, the following factors have a positive impact on the development of this market segment: growing environmental concerns, technological progress and increased production capacity. The global silicone coatings industry is on a growth trend due to the increasing demand for eco-friendly coatings around the world. The development of ecological construction around the world and growing concern for the environment, as well as increasingly stringent regulations regarding volatile organic compounds (VOCs) are supporting the development of this industry. At the same time, progress in technology and the development of the automotive industry will drive the transport and automotive industry. As the population in developing countries grows, infrastructure development is expected to provide car manufacturers with significant growth potential. In response to the above factors indicating the growth of the industry, silicone manufacturers are increasing their production capacity and infrastructure to meet the demands of the growing market. Moreover, major players in the coatings industry are engaging in partnerships and acquisitions to confirm the uninterrupted supply of raw materials. Joint ventures are an essential part of this industry and help companies to support their market position with long-term stability.

Undoubtedly, coating materials intended for many innovative, demanding applications should have superhydrophobic properties and very good thermal stability at the same time. An example of such a coating material is a porous superhydrophobic silica film, which is stable at high temperatures (~400 °C) and synthesized via the sol-gel method [18]. The described coating material has very good properties, but the area of application is limited due to the porous nature of the obtained coating, which results from the use of silica film. Coating materials with a wider range of applications can be obtained using polysiloxanes. Li et al. [19] obtained a durable and self-healing superhydrophobic coating with high thermal stability and long-term corrosion resistance obtained from a fluorine-free suspension containing methylsilicone polymers, modified SiO_2_ particles, graphene oxide and lamellar mica powder. There is information in scientific publications about research results indicating key factors influencing the achievement of good thermal resistance or superhydrophobic properties [20]. However, there are no publications analysing the factors influencing the simultaneous obtaining of those properties of silicone-containing composites that are very important in many of their applications, especially as coating materials for emerging applications.

The aim of this review is to analyse the impact of key factors on the simultaneous unique surface properties and thermal resistance of silicone-containing composites based on a literature review and the most important patent databases. The unique properties of organosilicon polymers result from the structure of the silicon atom and are related not only to the structure of the polysiloxane chain but also to the presence of functional groups and the place of their incorporation into the chain [20]. Undoubtedly, the most important factors influencing these properties are those related to the structure of organosilicon polymers and the presence of micro- and nanoadditives, as well as the modification methods used in the composite production process [21,22].

Therefore, this review will discuss the influence of factors related to the structure of organosilicon polymers, micro- and nanoadditives and methods of modifying and manufacturing composites containing silicones on the thermal and surface properties of these composites. The influence of nanoadditive dispersion obtained due to the presence of different functional groups and different methods of producing these composites will also be discussed. Methods for achieving simultaneous thermal stability and surface superhydrophobicity in silicone-containing composites will be analysed based on publications and patents.

## 2. Method of Conducting a Literature Review

The literature review of the last 20 years was performed taking into account the following keywords: thermal resistance, surface properties, silicone-containing composite, self-cleaning and silicone application and silicone emerging applications.

**Figure 1:**
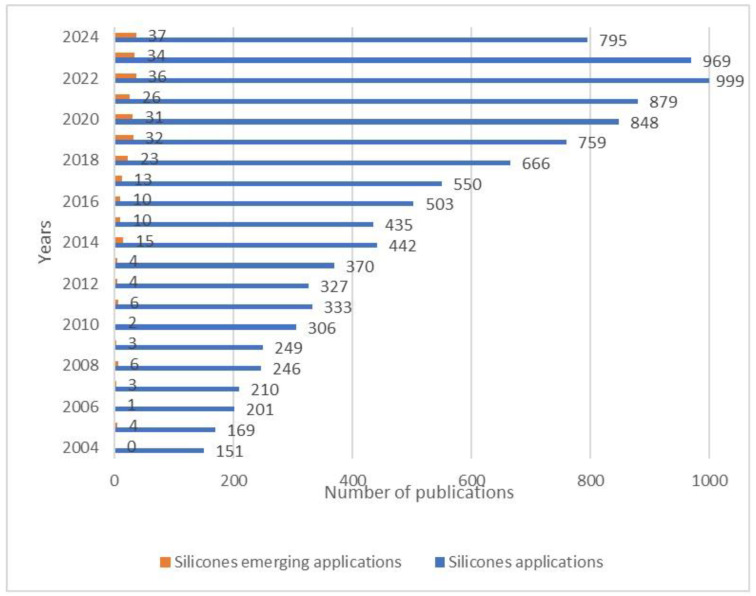
Number of publications on silicones applications and silicone emerging applications in 2004 to 2024 (based on Web of Science data).

Trends in the number of publications on silicone applications and silicone emerging applications from 2004 to 2024 based on data from the Web of Science (Figure 1) show a clear increase in the number of publications on emerging applications from 2014 to 2024. In contrast, only a few such publications were recorded per year from 2005 to 2013. The above data indicate a clear trend of growing interest in this topic.

**Figure 2:**
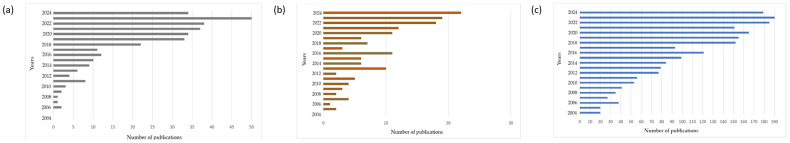
Number of publications on (**a**) silicone superhydrophobicity, (**b**) silicone fire resistance and (**c**) silicone thermal stability in 2004 to 2024 (based on Web of Science data).

The detailed trends in the number of publications on silicone superhydrophobicity, silicone fire resistance and silicone thermal stability (Figure 2a–c).

The detailed trends in the number of publications clearly confirms the trends in research interest in the topics discussed.

The research also covered Espacenet, Patentscope and Google Patents, taking into account the years 2020–2024, which resulted in the presentation of selected patents relevant to the subject. For example, as a result of the Google patents search, we received 671 results. However, the vast majority of patents focused only on superhydrophobic and self-cleaning properties without taking into account thermal resistance. Several patents were selected, concerning both the surface properties and thermal resistance of composites containing silicones. Patents selected based on the most promising applications will be discussed in the content of the publication. This review does not cover the use of flame retardants, especially halogenated ones. The use of such additives is limited in European Union countries and the USA on the basis of legal provisions (e.g., the registration, evaluation, authorisation and restriction of chemicals (REACH) regulation and the restriction of hazardous substances (RoHS) directive).

## 3. Factors Related to the Structure of Organosilicon Polymers

Obtaining polymer materials that have both very good thermal resistance and superhydrophobic properties is possible only for a few base polymers, which include fluoropolymers and polysiloxanes. The latter are much more commonly used due to their price, availability and processing possibilities. The unique properties of polysiloxanes result from the structure of the silicon atom, which influences the structure of the polysiloxane chain. The electron configuration of [Ne]3s^2^3p^2^ allows for a variety of interactions depending on the properties of other atoms constituting the molecule. In polysiloxanes, the basic interactions considered concern Si–O siloxane bonds. Certain features of this bond may result from dπ-pπ interactions between silicon and oxygen atoms [21]. However, other theoretical calculations indicate a small contribution of electrons from d orbitals [22]. Therefore, the currently prevailing model assumes a strongly ionic character of the Si–O bond and negative hypercoupling (p(O) → σ*(Si–X))_n_ where X is any atom bonded to silicon. These interactions are particularly strong when X is highly electronegative, e.g., O, F, Cl. It should be emphasized, however, that the concept of p–d interactions has not been completely rejected because theoretical calculations do not provide clear information about the nature of the Si–O bond [21]. To further elucidate the problem, Weinhold and West [23] performed ab initio calculations combined with natural bond orbital analysis techniques. These calculations enabled us to elucidate the fundamental differences between SiO and CO bonds that affect the molecular structure, vibrational rigidity and chemical basicity in polysiloxanes and organic ethers. It was found that the key to the enhanced siloxane X–O hyperconjugation lies in the larger X–O–X bond angle for X=Si. The obtained computational results have important implications for many aspects of silicon chemistry and polysiloxane chain structure, as well as for a better general understanding of basicity and hydrogen bonding phenomena in terms of the resonance-type (and not “electrostatic”) donor-acceptor concept. The unique structure of the polysiloxane chain resulting from the large Si–O bond length and the angles between chemical bonds of 112° for C–Si–C bonds, 143° for Si–O–Si and 110° for O–Si–O is responsible for the exceptional flexibility and possibility of rotation around the Si–O–Si bond of this chain. The homolytic strength of the siloxane bond results from the synergy of its partial ionic character and the double bond, since both effects increase the bond strength between the participating silicon and oxygen atoms. As a result, the Si–O bond can withstand higher temperatures than bonds commonly found in all-organic polymers, and polysiloxanes as a class exhibit higher thermal stability than their C–C counterparts. According to Dvornic [24], the above structural features cause the following properties of polysiloxane macromolecules:

- chain structure in the form of regular helices with substituents directed outwards and towards neighbouring chains,
- low intensity interactions between neighbouring organic substituents,
- effective shielding of the backbone by these substituents undergoing free rotation about their Si–C bonds,
- relatively large free volume between neighbouring chain segments resulting from the distinct mobility of substituents,
- significantly reduced possibility of interchain interactions resulting in small interpenetration of neighbouring chains and reduced probability of entanglement up to a high degree of polymerisation.

**Figure 3:**
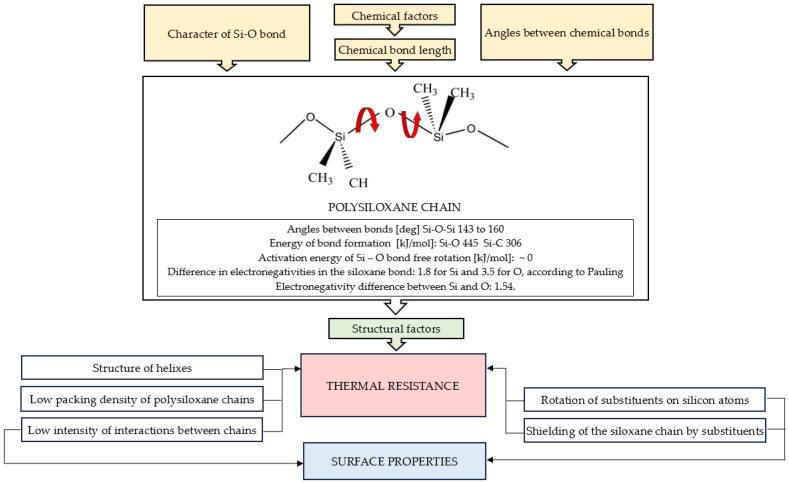
Structural factors affecting macroscopic properties of polysiloxanes.

The above-mentioned properties of macromolecules obviously translate into the macroscopic characteristics of polysiloxanes, as shown in Figure 3.

Thermal resistance with simultaneous surface hydrophobicity are some of the basic properties of polysiloxanes that determine their wide application possibilities. For example, the upper-limit temperatures for the onset of irreversible degradation of polysiloxanes may reach over 300–350 °C, while degradation temperatures of organic –C–C– type polymers can rarely exceed 150–200 °C [23]. The very good thermal resistance is due to the structure of the polysiloxane chain in the form of regular helices, which are additionally protected by outwardly directed substituents at silicon atoms. Such a substituent arrangement is possible due to the easy rotation of the polysiloxane chain around the siloxane bond. At the same time, the possibility of rotation of the substituents, which are most often alkyl groups, affects the hydrophobicity of the surface. As shown in Figure 2, the discussed properties are also influenced by small interactions between the polysiloxane chains and low packing of neighbouring segments.

Organic substituents at the silicon atom in the polysiloxane chain have a significant influence on both thermal resistance and hydrophobicity. It should be noted, however, that organic substituents, depending on the structure, have a varied influence on the properties of composites, as indicated by their ranking in accordance with increasing thermal resistance: C_2_H_5_ > CH_3_ > CH_2_ = CH > Cl_2_C_6_H_3_ > Cl_3_C_6_H_2_ > ClC_6_H_4_ > C_6_H_5_ [25].

The properties of polysiloxanes can also be modified using the grafting method, which is particularly effective for obtaining innovative coating materials. The preparation of polysiloxane grafted with poly(vinyl acetate) (PVAc) made it possible to obtain easy-to-clean coating materials alternative to fluoropolymer coatings [26]. Via free-radical polymerization of vinyl acetate and low-molecular-weight poly(dimethylsiloxane (PDMS), a number of materials with different comonomer contents were obtained. PDMS grafted with 20% PVAc showed similar parameters to poly(tetrafluoroethylene) (PTFE) coating with an analogous wetting angle value (99° vs. 100°) and surface energy (21.77 vs. 22.08 mJ/m^2^).

## 4. Factors Related to Micro- and Nanoadditives

The structure of organosilicon polymers has a very significant effect on the properties of the composites obtained with their participation. However, obtaining composites characterized by performance parameters meeting the requirements of innovative applications is possible only with the appropriate selection of micro- and nanofillers. In order to obtain properties corresponding to particularly high utility parameters, carbon nanotubes, montmorillonite and halloysite are used, and depending on the desired properties, others are selected for specific applications, such as boron nitride and zinc oxide. The range of possibilities is very wide. At the same time, it should be emphasized that in individual types of fillers, a number of types can be distinguished depending on the method of their production, which determines the size and structure of particles, and the method of modification, which affects the type of functional groups on the surface. A very good example is silica used as a standard filler.

Tong et al. [27] investigated the influence of the morphology and surface chemistry of different types of silica on the thermal and mechanical properties of the obtained silicone rubbers. High temperature vulcanized silicone rubber composites were prepared using colloidal silica (FSi), precipitated silica (PSi) and modified precipitated silica (MPSi) as reinforcing fillers. These fillers showed different effects on the properties of silicone rubber composites, including curing behaviour, mechanical properties at room temperature and low temperature, thermo-oxidative stability and solvent resistance. The difference in the effect is mainly due to the type of filler, surface area, Si–OH group content, amount of filler used and the method of surface modification, which causes different compatibility and interaction. Silicone rubbers containing FSi have the best mechanical properties (tensile strength, Young’s modulus), dynamic mechanical properties (the dynamic modulus (*E_0_*) and the glass transition temperature (*T_g_*)) and solvent resistance among the three types of reinforced silicone rubbers due to having the largest surface area and Si–OH group content. In contrast, the silicone composite containing 80 phr MPSi has the highest tear strength due to the best filler compatibility and dispersion. In addition, surface modification in MPSi reduces the acidity of this filler, which results in better resistance to thermal aging by reducing acid-catalysed degradation reactions.

Chen et al. [28] carried out a systematic study of the microscopic mechanisms of the effect of silica particle size on the properties of silicone composites. Five types of silica particles with average particle sizes of 20, 50, 100, 200 and 500 nm were selected. The surface of all fillers was modified with silane coupling agents. It was found that when the silica filler content was constant, the ultimate strength, fracture toughness and tensile strain at the fracture of the composite showed a monotonic increase with decreasing particle size. In the composite containing monodisperse submicroparticles, the improvement in strength results from the strong bonding at the interface between the particles and the polymer matrix. In addition, the submicroparticles hinder crack propagation. The improvement in hardness results mainly from the reduction of the number of cracks around the particles. In the composite containing nanoparticles, both the strong interface and the effect of hindering crack propagation contribute to the increase in composite strength. Moreover, the hierarchical structure of the network consisting of fillers of different particle sizes and bonded polysiloxane chains provides the composite with improved strength compared to composites containing particles of the same size. As a result, the composite filled with small nanoparticles capable of forming aggregates of different sizes exhibits significantly improved mechanical properties compared to the composite filled with submicroparticles.

Similar relationships were obtained using alumina as a filler. Three types of binary mixtures of alumina particles with different diameter ratios at constant total volume were used to reinforce the polysiloxane composite. The results showed that the thermal conductivity of the rubber filled with larger particles introduced in an amount below 50 wt.% was better than that of the rubber filled with smaller particles [29]. The obtained results further indicated that the polysiloxane composite containing dual-sized alumina nanoparticles exhibited improved thermal conductivity, tensile strength and reduced dielectric constant as compared to the composite containing single-sized nanoparticles. It was found that the key factor determining thermal conductivity is the creation of effective heat conduction paths due to the reduced thermal contact resistance at the filler–matrix interface. Moreover, surface modification of Al_2_O_3_ particles can increase the thermal conductivity of the composite. In the studies, 3-methacryloyloxypropyltrimethoxysilane was used as a silane coupling agent in the amount of 2 wt.%.

**Figure 4:**
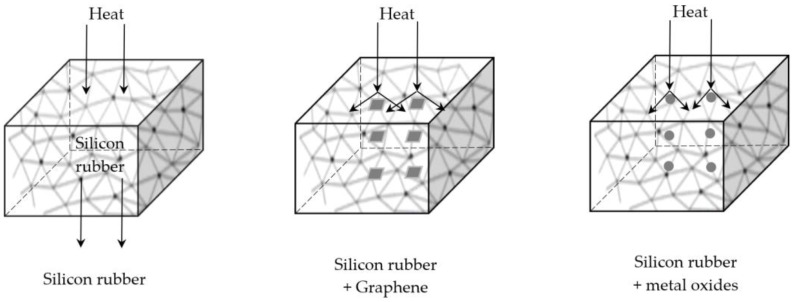
The effect of nanoadditives on thermal resistance of SR nanocomposites.

Similar studies have been conducted on the effect of particle size and the amount of micro- and nanofillers used, such as boron nitride (BN) and silicon carbide (SC), on thermal conductivity, the thermal expansion coefficient and thermal stability [30]. It was found that the introduction of various types of fillers with different particle sizes into polysiloxane composites has a smaller impact on thermal stability compared to the influence of the doping level. Thermal properties improve with an increase in the amount of introduced fillers. Kong et al. [31] investigated the effect of the amount of introduced nanofillers such as nano boron nitride BN, nano silicon nitride SN and synthetic nanodiamond ND on the thermal and mechanical properties of polysiloxane composites. Nanofillers were introduced in the following amounts of 0.5, 1.0, 1.5 and 2.0 vol.%. The composites were prepared via the casting moulding process. The introduction of thermally conductive nanoparticles into the polysiloxane matrix improved the thermal conductivity of the composites, which was associated with the increased crosslinking through physical and chemical interactions of the nanofillers with the polysiloxane matrix. BN particles have the most pronounced effect on thermal conductivity compared to SN and ND particles at any amount of the introduced filler. TGA studies showed that the thermal stability of the SN/silicone rubber system increased with the increase in the amount of the filler introduced, however, BN and ND acted as catalysts for the thermal degradation process of polysiloxane nanocomposites. This unfavourable effect was observed especially for those nanofillers that were introduced in the amount of 1.5 and 2 vol%. At the same time, homogeneous dispersion of nanoadditives such as graphene or metal oxides in the polymer matrix increases thermal resistance due to the dispersion of heat flux on these nanoparticles; see Figure 4.

The polymer/nanocomposite system is usually characterized by significantly higher mechanical or thermal properties than conventional polymer composites prepared with microfillers [32]. Nanofillers are characterized by a large specific surface area, which means that there may be a very large number of places where the filler interacts with the polymer matrix (interfaces), which are important for controlling the properties of nanocomposites. However, this number depends on the degree of dispersion of the nanocomposites. The greater the dispersion of polymer nanocomposites, the greater the number of interfaces. It should be noted, however, that in each case of using a nanoadditive, it is necessary to verify the effect of the additive on selected properties of the composite.

The presence of various functional groups on the surface of their particles is essential for obtaining a good degree of dispersion of fillers, especially nanofillers [33]. The use of fillers with well-selected functional groups on the surface of particles improves heat transfer and resistance of silicone composites to high temperatures, which is crucial in applications requiring thermal stability. Functional groups on the surface of nanoadditives can improve the resistance of composites to aggressive chemicals, which is important in many industrial applications. Due to better dispersion of nanoadditives and interaction with the silicone matrix, composites can show higher resistance to oxidation.

The presence of functional groups in silane coupling agents has a significant influence on the properties of polysiloxane composites [34]. Hydroxyl groups (–OH) improve the adhesion and interactions between nanoadditives and the matrix. Amino groups (–NH_2_) facilitate chemical reactions with the silicone matrix, increasing mechanical strength, and carboxyl groups (–COOH) improve the dispersion and stability of nanoadditives in the matrix.

The results of the work carried out by Jin et al. [35] indicate that the possibility of hydrogen bonds occurring between hydroxyl groups of, e.g., silica and hydroxyl/carboxyl/amide groups of the nanocomposite surface such as “n” carbon dots (CDs) may affect the dispersion of the nanocomposite in the matrix. Iron(III) oxide-hydroxide or ferric oxyhydroxide (FeO(OH)) and iron oxide (Fe_2_O_3_) have been used as high-temperature resistant additives for fluorosilicone rubber composites [36]. Tests carried out under aging conditions of 250 °C for 14 days showed that the combined use of various high-temperature-resistant additives improved the thermal stability of fluorosilicone rubber [37,38]. However, in the case of the main mechanical properties, such as tensile strength and elongation at break, the FeO(OH)-modified matrix showed better mechanical properties after ageing than before ageing.

Zhang et al. [39] showed that the tensile strength of a type of silicone resin with a unique structure/silicone rubber (MQ/SR) composites increased with the increase in MQ from 0 to 20 wt%. They attributed the obtained results to the increase in crosslinking density, which could be the effect of the reaction of the hydroxyl group of MQ with the terminal hydroxyl group of poly(dimethylsiloxane)/MQ (PDMS/MQ) acting as a bridge between the reduced graphene oxide (RGO) and rubber matrix so that the compatibility of RGO with polysiloxane is much better, and such effective interfacial interactions between RGO and PDMS promote load transfer from the rubber matrix to the nanofiller. This process improves the tensile strength of RGO/MQ/SR composites [40].

Boron nitride nanoplatelet-multilayer graphene (BN-MG) composites with silicone rubber (SR) (BN-MG/SR) possess functional groups that can contribute to the formation of greater interfacial adhesion between BN-MG fillers and the SR matrix via covalent bonding or van der Waals forces. These processes help reduce interfacial resistance and generate more thermal conduction paths and networks [41,42,43]. Interestingly, with increasing BN-MG loadings, the boundary between fillers and the SR matrix becomes blurred, which also affects the increased number of heat conduction paths. The presence of oxygen-containing functional groups, such as hydroxyl, on the surface of both BN and MG causes them to be negatively charged. As a result of poly(dimethyldiallylammonium chloride) (PDDA) functionalization, the zeta potential of BN is significantly increased and has a positive value, which confirms that PDDA with multiple positive charges successfully wrapped onto the BN surface, making the BN surface positively charged. The increase in the absolute value of the Zeta potential indicates that BN can be dispersed more uniformly and stably in the aqueous solution after functionalization by PDDA [43,44].

**Figure 5:**
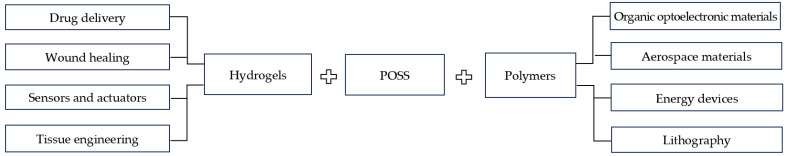
POSS as important building blocks used for construction of nanostructured composites.

Yu et al. [44] developed a dynamic silicone elastomer with two kinds of interactions prepared by incorporating a “carboxyl-amine ionic bond” and a “carboxyl-Al^3+^ coordinated ionic bond”. The carboxyl groups were bonded with amine groups and Al^3+^ ions, thereby forming common ionic bonds and coordinated ionic bonds, respectively. The formed carboxyl-Al^3+^ interactions can effectively improve the mechanical properties of this material, while the dense common ionic network between the carboxyl and amine groups can maintain the crosslinking integrity of the dynamic network. In order to effectively increase thermal stability and thermal aging resistance, silicone rubber (SR) was mixed with 2-aminoterephthalate, oxygen (2−), zirconium (4+) and tetrahydroxide (UiO-66-NH_2_). The results showed that even small doses (0.25–1.5 parts) of UiO-66-NH_2_ additive can effectively increase the thermal-oxidative stability of SR, effectively inhibit the degradation of SR chains and hinder heat transfer due to low thermal conductivity [45]. However, the introduction of the -NH_2_ group does not always give the best possible effect in terms of improving the properties, e.g., thermal, of the composite. The introduction of vinyl POSS (polyhedral oligomeric silsesquioxane) to SR gives a composite with better thermal parameters than the introduction of POSS with amino or chlorine functional groups. POSS with vinyl groups can crosslink with SR and can covalently bind to SR to form a denser three-dimensional network structure to introduce a more heat-resistant POSS cage structure. The modified SR provides improved thermal protection and insulation by transforming the POSS into a ceramic structure in situ to block heat and oxygen and by breaking more covalent bonds to absorb large amounts of heat under high temperature and ablation conditions [46,47]. The above described application of vinyl POSS for SR is just an example of the very wide range of possible applications. Generally speaking POSS are important building blocks used for the construction of nanostructured, organic-inorganic innovative polymeric materials, which can be applied in advanced materials; see Figure 5.

Intra- and intermolecular interactions due to the formation of hydrogen bonds are strengthened by the introduction of carboxyl groups into the composition of polymer molecules. However, research results have shown that components that allow for the creation of space or division between groups are also of significant importance for stability. Thus, polysiloxanes with carboxyl groups, having a distance between the siloxane skeleton and the carboxyl groups shorter than –CH_2_–CH_2_– or –CH_2_OCH_2_– decompose, while those with longer distances remain stable [48,49].

Graphene oxide (GO), which exhibits good hydrophilicity due to the presence of a large number of hydroxyl and carboxyl groups on its surface, is responsible for the poor interfacial compatibility of GO with the SR matrix. Therefore, it is necessary to add a coupling agent to adapt the hydrophilic surface of GO to the hydrophobic surface of SR. A silane coupling agent is often considered as a suitable functional molecule for GO due to its high hydroxyl content [50,51,52,53]. Vinyl-GO has a hydrophobic surface and probably good interfacial compatibility with SR, which, according to literature reports, is responsible for good interfacial compatibility in organic-inorganic composites and high enhanced thermal conductivity and thermal stability [51,52,53,54].

Hu et al. [55] utilized dynamic reversible carboxylamine bonds to develop rapidly self-healing and highly thermally conductive Al_2_O_3_@SR/SCNR (slightly crosslinked natural rubber) composites. Dynamic and reversible crosslinking between carboxyl and amine functional PDMS and different sizes of spherical Al_2_O_3_ fillers were used and introduced into the SR system. The dynamic ionic reaction between carboxyl and amine functional PDMS was considered as the source of high self-healing efficiency, while the introduction of alumina improved the thermal conductivity of the composites and accelerated the heat transfer rate.

**Figure 6:**
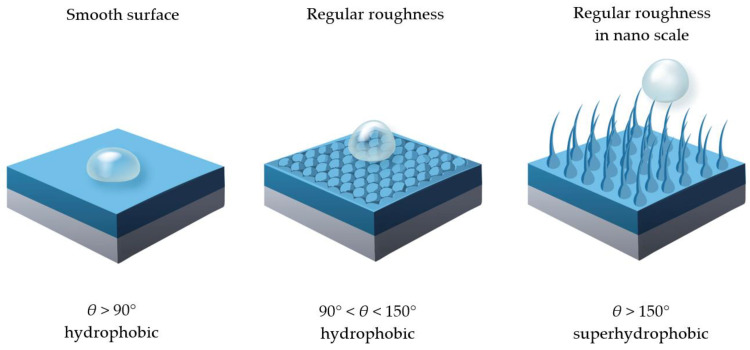
The effect of surface structure on the contact angle.

The proper selection of fillers also plays a key role in the production of polysiloxane composites with surface properties consistent with their application. In recent years, there has been significant progress in the production and application of superhydrophobic coating materials [56]. The surfaces of such materials are characterized by a designed regular texture and a defined chemical structure of the surface, depending on its desired properties [57]. The inspiration for designing surface textures are often found in nature, such as, for example, lotus or rice leaves, butterfly wings, shark skin and many others (Figure 6).

The production of such surfaces is a complex process, and the obtained results are influenced by the appropriate selection of the polysiloxane structure and the method of its crosslinking as well as the appropriate selection of fillers, often containing nanoparticles. One of the important parameters necessary to obtain a superhydrophobic surface is its regular roughness in the nanoscale, which, according to the Cassie model, creates a composite surface with a very regular structure [58]. Air is occluded in the irregularities of such a surface, which reduces the wettability and creates a superhydrophobic surface, as shown in Figure 6.

In addition, the application parameters defining the properties of such a surface are a high apparent wetting angle (*θ*_C_ > 150°), low wetting angle hysteresis (*θ*_C_ < 10°) and a low slip angle (*θ*_C_ < 5°). The superhydrophobic surfaces discussed find a number of different applications for the protection and securing of building materials as so-called self-cleaning surfaces [4], surface protection of electrical insulators [59] or other precision devices [60].

An interesting case is also the composites characterized by the presence of hollow micro/nanospheres, also known as micro/nanoshells, micro/nanocapsules or micro/nanoballoons (HSN, hollow silica nanospheres) [61]. The hollow spheres contain a single large cavity, inside which the outer diameter varies from 0.1 to 1000 µm [62], and the walls are about 1–10% thick in terms of their diameter [61]. These structures are characterized by low density, a low dielectric constant, low thermal conductivity and good sound absorption, dispersion, fluidity and stability [63]. Some of the studies on epoxy matrix composites indicate that additional reinforcement is possible by using HSN. An example is the work of Yung et al. [63], who obtained a composite characterized by thermal conductivity lower by 54%, *T_g_* temperature higher by 38 °C and dielectric constant decreasing with the increase of the empty glass microsphere.

## 5. Factors Related to Composite Manufacturing Methods

**Figure 7:**
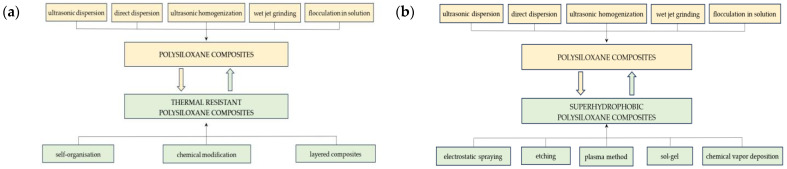
(**a**,**b**) Methods of manufacturing polysiloxane composites: (**a**) thermal resistant, (**b**) with superhydrophobic surfaces.

The method of its production has a significant influence on the properties of the composite, which is mainly related to the degree of dispersion of fillers, especially nanofillers. The most commonly used methods of producing composites are direct or ultrasonic dispersion, flocculation in solution, ultrasonic homogenization and wet jet milling [36]. Good dispersion of fillers improves the thermal resistance of nanocomposites. Obtaining superhydrophobic properties usually requires the use of more precise and specific methods, such as etching [64], sol-gel [65], electrostatic spraying [66], chemical vapor deposition [67] and the plasma method [33]. Therefore, the choice of processing methods depends on the parameters that the polymer composites are to meet, which is schematically shown in Figure 7a,b.

As can be seen in the above diagrams, obtaining composites with increased thermal resistance requires mass processing methods other than in the case of superhydrophobic surfaces, where good effects can often be obtained via surface modification.

The use of specific methods for shaping the hydrophobic surface allows coatings to be obtained with the texture most suitable for the requirements of individual applications. However, the biggest problem is obtaining coatings with the appropriate strength. Superhydrophobic coatings often have low mechanical strength. Improvement of mechanical properties can be achieved by introducing appropriate fillers, e.g., nanosilica [68]. The resistance of superhydrophobic coatings to chemicals is also of great importance in innovative applications. A significant increase in the chemical resistance of such coatings can be achieved by appropriate selection of polysiloxanes [68,69]. The tests confirmed that the obtained coatings are resistant to acids, alkalis and long-term UV radiation. In addition, superhydrophobic coatings often degrade under the influence of changing environmental conditions. An effective solution to this problem is regenerative superhydrophobic coatings, which can regain their properties after degradation [68,70]. In conclusion, it should be stated that research conducted on improving the composition and methods of manufacturing of superhydrophobic coatings will significantly expand the possibilities of their application.

## 6. Analysis of Methods for Achieving Simultaneous Thermal Stability and Surface Superhydrophobicity in Silicone-Containing Composites

**Figure 8:**
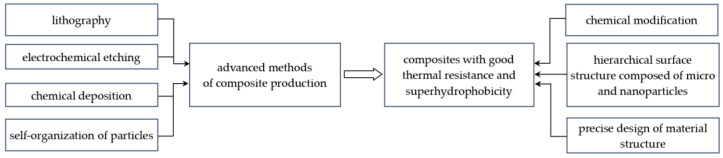
Integrated methods of manufacturing composites characterised by high thermal resistance and superhydrophobicity.

Based on the analysed available publications, it is possible to discuss the mechanisms of simultaneously obtaining thermal stability and surface superhydrophobicity in composites containing polysiloxanes. Simultaneously achieving thermal stability and superhydrophobicity in composites requires the use of both advanced materials and manufacturing techniques; see Figure 8.

The key factors for the production of polysiloxane composites with such properties are the hierarchical surface structure, chemical modification of the composite surface and integrated production methods. According to the Cassie model, the basic condition for obtaining a superhydrophobic surface is a three-dimensional, hierarchical surface texture connecting micro- and nanoscale domains facilitating air occlusion [58]. This structure of the surface layer helps minimize the contact of water with the material surface, leading to an apparent contact angle *θ_C_* > 140° created by the tangent to the surface of the drop with the substrate at the point of contact. The hierarchical structure of the surface consisting of micro and nanostructures can be obtained by using lithography, electrochemical etching, or chemical deposition, as well as via self-assembly of particles. An example of such a material is a coating made of a composite silicone-epoxy resin obtained via a two-stage spray coating method [71]. Such coatings have found application in photovoltaic panels, light-emitting diode panels and glass curtain walls, among others. It should be emphasized that achieving both properties in one material requires the use of integrated production methods combining different techniques. One of the effective methods of obtaining composites characterized by good thermal stability and superhydrophobicity is the production of layered composites, in which different layers of materials are applied in a controlled manner, often using the ability of polymers to self-assemble. An example of such materials are epoxy-siloxane layered composites used as protective coatings, e.g., on polycarbonate [72]. Good results are also obtained using sol-gel techniques, which enable controlled modification of the material surface [73].

In summary, achieving simultaneous thermal stability and superhydrophobicity in composites requires precise design of the material structure, appropriate chemical modifications and advanced production techniques. The integration of these elements allows the creation of materials with unique properties that can be used in demanding environmental conditions.

Based on the analysis of a number of publications included in the text of the article, a table was prepared summarizing and systematizing the discussed information; see Table 1. The primary and structural factors influencing thermal resistance and superhydrophobicity are synthetically presented.

## 7. Conclusions

Based on a detailed analysis of the research results and the presented factors influencing the thermal resistance and surface properties of composites containing silicone, the following conclusions can be drawn regarding the most important key factors.

1. Structure of organosilicon polymers: One of the key factors determining the unique properties of these composites is the structure of the organosilicon polymer. The thermal properties are due to the presence of Si–O bonds, which are characterized by high thermal stability. The rotation of substituents around Si–C bonds along the polysiloxane chain limits the interactions between adjacent segments, which also affects the thermal resistance of the material.
2. Micro- and nanoparticle additives: Another important factor influencing the properties of composites is the selection of appropriate microfillers and nanoparticles. Additives such as aluminium oxide (Al₂O₃) or boron nanoparticles significantly affect thermal conductivity and mechanical properties, which allows the use of composites in more demanding applications.
3. Surface modification: Achieving simultaneous superhydrophobicity and thermal stability requires precise surface modification and appropriate selection of manufacturing methods. The use of methods such as the sol-gel technique, chemical deposition or lithography allows a surface structure to be obtained on micro- and nanoscale, which helps to minimize contact with water.
4. Integration of manufacturing methods: The best results in the production of composites with exceptional properties are achieved with integrated manufacturing techniques that allow for controlled deposition of successive layers of materials or independent assembly of polymers. An example is layered epoxy-silicone composites, which exhibit excellent protective properties.

The unique properties of these composites, such as high thermal resistance and superhydrophobicity, enable their wide use in various industries, including electronics, photovoltaics, medicine and construction. Further research should focus on improving the production methods in order to increase the mechanical and chemical stability of these materials.

Nowadays, silicones are practically indispensable in everyday life, being used as coating, insulat and waterproofing materials resistant to extreme weather conditions. Research teams around the world are working on new compounds and materials that meet the emerging market requirements. Functionalization of polysiloxanes with appropriate chemical groups and selection of active fillers depending on the planned applications, combined with innovative crosslinking and manufacturing methods, allow intelligent materials capable of responding to external stimuli to be obtained [74]. The properties of new types of silicones such as thermoplasticity, self-healing ability, surface activity, electromechanical activity and magnetostriction and thermo-, photo- and piezo-responsivity are particularly valuable [75,76].

## Figures and Tables

**Table 1:** The primary and structural factors influencing thermal resistance and superhydrophobicity.

Factor		Response in Properties		Ref
Primary	Structural	Thermal	Superhydrophobic	
Chemical: - Si atom structure - Si-O bond length - S-O bond character	- Helical structure of PS chain	X		[21,22,23,24]
	- Low interactions between PS chains	X	X	
	- Low packing density of PS chains	X		
	- Rotation of substituents on Si atom	X	X	
	- Shielding of PS chain by substituents	X	X	
	- Size of organic substituents	X	X	[25]
	- Grafting of active substituents		X	[26]
Micro and nano additives: - Types of silica	- FSi, Psi, MPSi - different effects on the properties of silicone rubber composites, including low temperature, thermooxidative stability	X		[27]
- Silica particle size	- Composite filled with small nanoparticles forming aggregates of different sizes exhibits significantly improved properties compared to the effect of submicroparticles	X		[28]
- Binary mixtures of alumina particles	- Composite containing dual-sized alumina nanoparticles exhibited improved thermal conductivity	X		[29]
- Amount of micro- and nano-fillers used, such as BN and SC	- Different particle sizes of various fillers types has a smaller impact on thermal stability compared to the influence of the doping level	X		[30]
- Nano BN, nano SN and synthetic ND	- BN particles have the most pronounced effect on thermal properties compared to SN and ND particles at any amount	X		[31]
- Nanofillers characterized by large specific surface area and presence of functional groups	- Functional groups can improve the resistance to aggressive chemicals. Better dispersion of nanoadditives and interaction with the silicone matrix gives higher resistance to oxidation	X		[32,33,34]
- Hydrogen bonds occurring between hydroxyl groups of filler and silicone matrix	- Better dispersion of the nanoadditive in the matrix resulting in high-temperature resistance	X		[35,36,37,38]
- PDMS/MQ acting as a bridge between RGO and rubber matrix	- Effective interfacial interactions between RGO and PDMS promote load transfer from rubber matrix to nanofiller improving stability	X		[39,40]
- Formation of greater interfacial adhesion between BN-MG fillers and the SR matrix via covalent bonding or van der Waals forces	- Increased number of heat conduction paths	X		[41,42,43,44]
- Application of special additives such as UiO-66-NH_2_ or POSS with vinyl groups	- Effective inhibition of the SR chains degradation and hindered heat transfer due to low thermal conductivity. Transformation of POSS into a ceramic structure blocking heat and oxygen and absorbing large amounts of heat under high temperature and ablation conditions	X		[45,46,47]
- Formation of hydrogen bonds strengthened by the introduction of carboxyl groups into the composition	- Significant importance for stability when a distance between the siloxane skeleton and the carboxyl groups shorter than –CH_2_–CH_2_– or –CH_2_OCH_2_–	X		[48,49]
- Hydrophobic, functionalised GO containing vinyl groups	- Good interfacial compatibility in organic-inorganic composites and high enhanced thermal conductivity, thermal stability	X		[50,51,52,53,54]
- Dynamic reversible carboxylamine bonds developing rapidly self-healing and highly thermally conductive Al_2_O_3_@SR/SCNR	- Introduction of alumina improved the thermal conductivity of the composites and accelerated the heat transfer rate.	X		[55]
- Selection of PS structure and the method of its crosslinking and appropriate selection of fillers	- According to the Cassie model important parameters to obtain a superhydrophobic surface is its regular roughness in the nanoscale. Air occluded in the irregularities of such a surface reduces wettability and creates a superhydrophobic surface		X	[56,57,58,59]
- PS composites containing hollow silica nanospheres	- Increased thermal stability due to the structure of additive	X		[61,62,63]
Composite manufacturing methods - Direct or ultrasonic dispersion, flocculation in solution, ultrasonic homogenization and wet jet milling	- Good dispersion of fillers improves the thermal resistance of nanocomposites	X		[36]
- Superhydrophobic properties requires the use of more precise and specific methods	- Etching [64], sol-gel [65], electrostatic spraying [66], chemical vapor deposition [67] and the plasma method [33]		X	[33,64,65,66,67]
- Chemical resistance of superhydrophobic coatings can be achieved by appropriate PS selection	- Solution to this problem are regenerative superhydrophobic coatings, which can regain their properties after degradation	X		[68,69,70]
Simultaneous thermal stability and surface super-hydrophobicity in PS composites—Analysis of methods - Achieving thermal stability combined with superhydrophobicity in composites requires the use of both advanced materials and manufacturing techniques	- The key factors for the production of polysiloxane composites with such properties are the hierarchical surface structure, chemical modification of the composite surface and integrated production methods	X	X	[71,72,73]
	- According to the Cassie model, the basic condition for obtaining a superhydrophobic surface is a three-dimensional, hierarchical surface texture connecting micro- and nanoscale domains facilitating air occlusion	X	X	[58]

## Data Availability

Not applicable.

## Abbreviations

BN	boron nitride
BN-MG	boron nitride nanoplatelet-multilayer graphene
CAGR	compound annual growth rate
CDs	Carbon Dots
E_0_	dynamic modulus
FSi	colloidal silica
GO	graphene oxide
HSN	hollow silica nanospheres
MPSi	modified precipitated silica
MQ	a type of silicone resin with a unique structure
ND	nanodiamond
PDDA	poly(dimethyldiallylammonium chloride)
PDMS	poly(dimethylsiloxane)
POSS	polyhedral oligomeric silsesquioxane
PSi	precipitated silica
PVAc	poly(vinyl acetate)
PTFE	poly(tetrafluoroethylene)
*θ*_C_	wetting angle
REACH	registration, evaluation, authorisation and restriction of chemicals
RGO	reduced graphene oxide
RoHS directive	restriction of hazardous substances directive
SC	silicon carbide
SCNR	slightly crosslinked natural rubber
SR	silicone rubber
*T_g_* temperature	glass transition temperature
TGA	thermogravimetric analysis
UiO-66-NH_2_	2-aminoterephthalate, oxygen (2-), zirconium (4+), tetrahydroxide
VOCs	volatile organic compounds
